# Surgery to Strength: A Literature Review of Postoperative Rehabilitation for Hindfoot Surgeries

**DOI:** 10.7759/cureus.92640

**Published:** 2025-09-18

**Authors:** Ahmed Shalaan, Mohamed A Khalafallah, Ibrahim Moqbel, Islam S Elhois, Yahya A Mahmoud, Mohamed Hashem

**Affiliations:** 1 Trauma and Orthopaedics, Bedfordshire Hospitals NHS Foundation Trust, Bedford, GBR; 2 Smart Health Centre, OrthoGlobe, London, GBR; 3 Faculty of Medicine, Alexandria University, Alexandria, EGY; 4 Trauma and Orthopaedics, Faculty of Medicine, Cairo University, Giza, EGY; 5 Trauma and Orthopaedics, Qena General Hospital, Qena, EGY; 6 Trauma and Orthopaedics, Faculty of Medicine, Al-Azhar University, Gaza, PSE; 7 Orthopaedics, Frimley Health NHS Foundation Trust, London, GBR

**Keywords:** ankle, foot, hindfoot, physiotherapy, recovery, rehabilitation, subtalar, weight-bearing

## Abstract

Calcaneal osteotomy, subtalar fusion, and triple fusion are the main surgical interventions used to correct foot deformities, reduce pain, and restore functionality in arthritis, flatfoot deformity, and post-traumatic complications. The outcomes of these surgical interventions are dependent on careful surgical planning, postoperative patient compliance, and comprehensive multidisciplinary management.

Rehabilitation generally follows a stepwise approach, starting with non-weight-bearing and partial weight-bearing and ending with targeted physiotherapy. This will enhance mobility and strength and minimize complications such as muscle atrophy and joint stiffness. Patient compliance is critical to achieve adequate results as it greatly influences bone healing and leads to better functional recovery.

Patient demographics and comorbidities and specific surgical techniques often influence rehabilitation approaches and need individualized tailored treatment plans. This review presents updated evidence and recommends a standard postoperative rehabilitation protocol for these three surgical techniques. Additionally, it will discuss the variations between different protocols in clinical practice, highlight the causes of those differences, and propose recommendations for future research.

Recently, it has become increasingly clear that there is a lack of consistent, high-quality evidence to guide postoperative rehabilitation after hindfoot surgery. This highlights the growing need for large-scale research to define postoperative protocols, weight-bearing guidelines, and advanced therapeutic modalities. Establishing evidence-based, broadly accepted guidelines could significantly enhance healing outcomes, functional recovery, and patient satisfaction in foot surgery rehabilitation.

## Introduction and background

Calcaneal osteotomy, subtalar fusion, or triple fusion can be performed to correct deformities such as pes cavus, pes planus, hindfoot varus/valgus, or equinus, relieve pain, and improve function for patients with a variety of pathological conditions including arthritis, deformities, or post-traumatic complications [1-3]. These procedures involve the surgical realigning or fusing of foot and ankle bones to achieve the desired structural and functional outcomes.

The subtalar joint plays an important role in the foot biomechanics, which makes complex foot and ankle movements possible during our daily activities. Subtalar fusion non-union is a disabling condition that impairs patients' function and is a challenging situation for surgeons, particularly if associated with high-risk factors such as diabetes, smoking, revision surgery, history of infection, and peripheral vascular disease [2-4]. This complication can arise after surgical interventions like subtalar fusion, which is intended to stabilize the subtalar joint and treat pathological conditions such as arthritis or other foot deformities [5-7].

The development of subtalar fusion non-union may be influenced by pre-existing conditions such as high arch (pes cavus) or flatfoot (pes planus), which can cause abnormal weight-bearing distribution, leading to cartilage wear and potential arthritis [8-10]. Several surgical procedures may be used to address that particular complication, such as a revision procedure with bone grafting, calcaneal osteotomy to correct alignment, or adjuvant procedures (e.g., bone stimulators) in selected cases. These aim to restore physiologic foot and ankle biomechanics and improve overall foot function [1,4,6,11].

The rehabilitation protocols following calcaneal osteotomy, subtalar fusion, and triple fusion are critical for patient recovery and usually include initial immobilization followed by gradual weight-bearing. Also, understanding of the nuances of these deformities and the surgical interventions used for their correction is important for effective rehabilitation that optimizes functional recovery for those patients [11-14].

## Review

This article is a literature review aimed to synthesize evidence-based postoperative rehabilitation protocols for patients undergoing calcaneal osteotomy, subtalar arthrodesis, or triple arthrodesis. A systematic search was performed using electronic databases including PubMed, Cochrane Library, and Google Scholar. The search covered publications from 2001 through early 2025 to ensure both historical context and recency. To incorporate current clinical practices, institutional protocols were also obtained from the National Health Service (NHS) Trust intranets and specialized orthopaedic centers such as Chesapeake Orthopaedic and Sports Medicine Center, BayCare Foot and Ankle Center, Wye Valley NHS Trust, and South Bend Orthopaedics. These protocols were included not to replace peer-reviewed evidence but to reflect real-world practice. Highlighting them underscores the existing gap between commonly applied institutional guidelines and standardized, evidence-based recommendations.

The inclusion criteria encompassed English-language literature, clinical studies including randomized controlled trials, cohort studies, and case series, systematic reviews, meta-analyses, and official institutional rehabilitation guidelines. The focus was on sources detailing postoperative rehabilitation components such as weight-bearing progression, immobilization, physiotherapy exercises, and return-to-activity guidelines for the specified hindfoot procedures.

Sources were excluded if they focused exclusively on surgical techniques without addressing rehabilitation, involved animal or biomechanical studies, or were published in languages other than English. This approach ensured the inclusion of contemporary and clinically relevant information.

The identified sources were examined thematically to identify areas of consensus, established best practices, and controversies or evidence gaps requiring further exploration. This methodology allowed the synthesis of both peer-reviewed literature and institutional knowledge, providing a balanced overview of current rehabilitation practices and their limitations.

Thematic review and discussion

Similarities and Differences Across the Three Procedures

Calcaneal osteotomy, subtalar fusion, and triple fusion are distinct surgical interventions with overlapping goals but unique applications and techniques. All three procedures aim to correct deformities, alleviate pain, and restore functionality, usually after trauma, arthritis, or other foot and ankle pathologies. Calcaneal osteotomy often preserves joint motion and aims to realign the calcaneus with the hind and midfoot [15]. On the other hand, subtalar fusion and triple fusion are permanent arthrodesis; the former targets only the subtalar joint, while the latter includes the subtalar, talonavicular, and calcaneocuboid joints, making it a more aggressive procedure. Accordingly, triple fusion is typically reserved for severe deformities or advanced arthritis [16,17]. The primary difference lies in joint preservation, as osteotomy aims to maintain mobility, whereas fusions sacrifice it for better structural integrity and stability, particularly in patients at risk of progressive collapse, meaning irreversible loss of hindfoot alignment and stability. Despite these differences, all three require careful surgical decision-making, planning, and subsequent postoperative compliance for better results [18].

Postoperative Goals and Challenges

The primary postoperative goal is to ensure proper healing, maintain joint stability, and provide pain relief. This includes strict immobilization and non-weight-bearing (NWB) protocols to avoid fixation failure and malunion [15]. On the other hand, early interventions in physical therapy are critical to restoring mobility and reducing stiffness. However, achieving these goals is not easy to pursue in clinical practice due to factors such as comorbidities, degree of compliance, and the complexity of the surgery [18]. Early identification of complications such as wound dehiscence or infection is important to avoid delays in rehabilitation or recovery. Therefore, close monitoring in the early postoperative weeks can greatly affect the final outcome.

For calcaneal osteotomy, challenges like the degree of displacement needed to correct the deformity without soft tissue complications require precise planning and meticulous surgical technique to avoid neurovascular injuries or delayed wound healing [19]. Subtalar fusion is used to stabilize the subtalar joint, especially in cases of post-traumatic arthritis or complex deformities. Bone grafting and rigid fixation techniques might be of great help in achieving a sound stable fusion [16]. However, delayed union or non-union in subtalar fusion remains a significant concern, particularly in patients with poor vascularity or osteoporosis.

Rehabilitation following triple arthrodesis presents exceptional challenges due to the fusion of the talonavicular, calcaneocuboid, and subtalar, which fundamentally restricts foot adaptability. This demands prolonged immobilization alongside meticulous monitoring to prevent compensatory arthritis in adjacent joints, while patients endure extended recovery periods requiring custom orthotics for rigid-foot support and intensive gait retraining to navigate permanently altered mechanics. In addition, the healing of fusion sites often takes several months, which requires long periods of NWB, leading to muscle atrophy, stiffness, and unsatisfactory overall functional outcomes. Additionally, there is a risk of complications such as infection, wound healing issues, and hardware failure. Gait retraining is an integral part of all rehabilitation protocols after triple fusion, as fusing multiple joints alters normal foot mechanics and can lead to long-term functional limitations. Moreover, early physiotherapy and tailored rehabilitation plans are key to regaining strength, adapting to altered biomechanics, and improving outcomes [17].

Pain control is also critical due to the aggressive nature of these surgeries, especially with the need to balance pain relief and opioid overuse. Other patient factors, such as smoking, poorly controlled diabetes, and poor bone quality, can delay healing and increase the risk of complications. Subsequently, preoperative optimization of modifiable risk factors, such as glycemic control and smoking cessation, can enhance postoperative outcomes [8,11].

As such, the delicate balance between surgical precision, patient adherence, and comprehensive postoperative care highlights the need for a multidisciplinary approach involving surgeons, physical therapists, podiatrists, and other healthcare providers to address the goals, amplify the benefits, and reduce the risks of such complex surgical work. Patient adherence to postoperative protocols is a significant determinant of outcomes, as in hindfoot surgeries non-compliance with weight-bearing restrictions has been shown to increase the risk of complications, including non-union, by more than threefold [15]. It is of utmost importance to address patient education and expectations before surgery, as this can provide a sense of support to the patient, improve compliance, reduce anxiety, and contribute to a smoother recovery.

Role of Patient Compliance in Rehabilitation Outcomes

**Figure 1:**
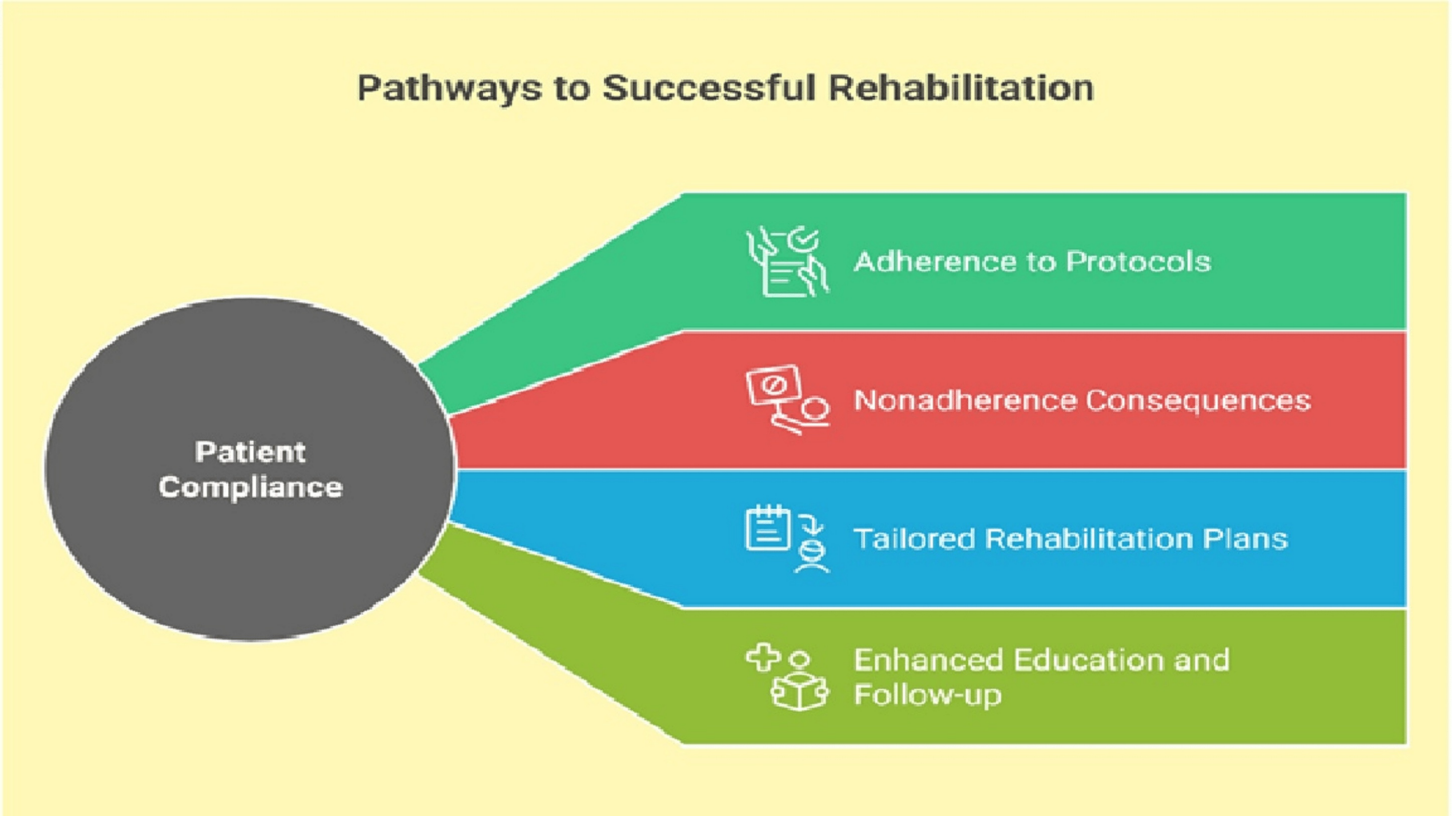
Role of patient compliance in rehabilitation outcomes Image Credits: Ahmed Shalaan

Strict adherence to postoperative protocols, including immobilization, physical therapy, and weight-bearing restrictions, is critical for achieving optimal functional outcomes following hindfoot surgeries, including calcaneal osteotomy, subtalar fusion, and triple fusion. Prospective cohort studies confirm that strict adherence to rehabilitation protocols significantly enhances joint stability (OR: 3.2; 95% CI: 1.8-5.7) and functional recovery (American Orthopaedic Foot and Ankle Society (AOFAS) score Δ +24.5 points), whereas non-compliance independently predicts suboptimal outcomes, including surgical failure requiring revision [15]. Moreover, individually adapted rehabilitation programs are more effective in motivating patients, who comply better and have better outcomes [18]. Other factors such as patient motivation and psychosocial support can improve compliance. Also, implementing simple strategies such as setting achievable goals, patient education, regular follow-ups, and maintaining good communication can improve patient engagement and recovery [19] (Figure 1).

The recommended postoperative rehabilitation protocol

The First Phase

Protocols for early postoperative care following foot surgeries share several common factors in the first phase, which extends from two to six weeks; these commonalities are critical for patient recovery and rehabilitation. The NWB period is a critical feature, as almost all the protocols have established a strict NWB period during the first phase [20-23]. This restriction is necessary to promote the healing of bone as well as soft tissues with minimal stress over the surgical site. Furthermore, swelling management is universally aggressive, with foot elevation being a main practice across protocols to treat early postoperative oedema. It is also important to control inflammatory and pain processes, although this is a very controversial aspect, with the prescription of analgesics and anti-inflammatory agents, which is essential for patient comfort and treatment success [24,25]. Cold therapy, including ice packs or other cooling devices, is often advised in addition to elevation and medications to reduce inflammation and swelling in the early postoperative stages. In addition, all protocols include therapeutic exercises directed towards strengthening the hips and knees, as well as the upper extremities and core muscles, to maintain fitness levels during the NWB phase. Range of motion (ROM) exercises are sometimes encouraged with care to prevent excessive dorsiflexion or eversion to maintain joint mobility [20,26]. However, supervision during early ROM exercises is often advised to minimize overloading the healing structures while preserving joint flexibility.

Progression to weight-bearing is another shared thread, with protocols gradually transitioning from NWB to partial or complete weight-bearing. This often includes using removable boots and/or a weight-bearing-as-tolerated (WBAT) approach after the initial healing [20,27]. The timing of this progression relies on several factors, including the nature of the surgery, X-ray findings, and patient tolerance. Protocols often recommend starting with partial weight-bearing, under supervision, before going to full weight-bearing to minimize the risk of non-union or hardware failure.

Finally, all protocols require more research to establish a consensus for the optimal timing for transitioning from NWB to weight-bearing activities, as well as the best practices for ROM exercises that balance preventing stiffness while ensuring adequate healing. Current variations between protocols underscore the importance of high-quality, well-designed comparative studies that can result in evidence-based recommendations for weight-bearing advancement following foot and ankle procedures. Moreover, we should consider patient-specific factors, such as bone density, age, comorbidities, and overall functional status, that are likely to achieve the best outcomes during this phase. After initial healing is achieved, the majority of protocols encourage moving to the second phase, with a gradual return to regular weight-bearing and other activities.

The Second Phase

The current protocols for the second phase of recovery after surgery recommend starting between two and 10 weeks postoperatively, with most extending the phase to 6-12 weeks. There is significant variation in the duration of this phase, but the average time generally falls within 6-12 weeks after surgery. Gradual weight-bearing during this phase is mainly consistent across most protocols, with minor timing and intensity variations [14,28,29]. Some protocols recommend progressing from 25% to 100% weight-bearing in a walking boot by week 10, while others allow for WBAT in a controlled ankle motion (CAM) boot [27,30,31]. Recent guidelines suggest a transition from partial to full weight-bearing, but the timing of this progression varies among the different protocols [31,32]. Further research is needed to establish the optimal and safest timeline for weight-bearing activities.

ROM exercises are universally endorsed, although the timing for initiating these exercises differs [33]. Both active and passive exercises are emphasized, while excessive eversion or dorsiflexion should be avoided [32]. In contrast, protocols for calcaneal osteotomy may permit earlier gentle ROM exercises for adjacent joints, provided the surgical site remains protected [18,27]. Maier et al. reported long-term results after triple fusion and drew attention to possible stiffness in adjacent joints if ROM was overly delayed, underlining the importance of a timed approach to ROM [33]. Gradual progressive ROM exercises are recommended to avoid disrupting healing joints and prevent postoperative stiffness and loss of function. Moreover, the initiation of early ROM activity in selected patients may help in preventing the development of scar tissue and adhesions; however, this requires careful clinical judgment.

Some protocols include specific exercises, such as alphabet movements and calf pumping [21]. Others recommend beginning gentle, active ROM exercises only after two weeks. Whatever the method used, there is a consensus that the restoration of motion is important [27,34]. Research comparing early versus delayed initiation of ROM exercises could help clarify the most effective approach. Strengthening exercises are similarly standardized across protocols. Recent guidelines incorporate intrinsic foot strengthening and bilateral balance exercises, while others emphasize mini-squats and leg presses. Some protocols focus on strengthening the hip and knee [32]. Scar mobilization is also a routine suggestion in other protocols, using soft tissue massage and scar mobilization techniques [21,32]. Aspirin and vitamin D analogs can prevent thrombosis and improve bone healing; yet further research is still needed to confirm their safety and efficacy [35,36].

The use of protective devices is an important aspect of these protocols. Some recommend CAM boots for weight-bearing progression, while others prefer surgical boots [27,30,37]. Recent studies have also utilized ankle braces [21,38]. Sheean et al. reported that custom orthoses and ankle braces, when combined with structured rehabilitation programs, were associated with improved functional scores and pain reduction following ankle and subtalar fusions [38]. Future investigations could compare patient outcomes to evaluate which device provides optimal support, pain control, and function. More research is needed to clarify the role of the Biomechanical Ankle Platform System (BAPS) in proprioceptive training. This device is specifically designed to enhance balance and joint awareness during the later phases of rehabilitation, particularly in patients recovering from hindfoot surgeries [21]. Protocols that include aquatic therapy highlight its benefit in enabling low-impact gait training during early weight-bearing, particularly for patients with limited tolerance for land-based exercises [22,27].

A uniform definition of progression criteria across protocols and the inclusion of objective parameters, like pain scores, would lead to more consistent outcomes. Once the patient has become confident with basic mobility and partial weight-bearing in the second phase, rehab gradually begins to shift towards more dynamic strengthening, balance work, and real-world function in phase 3.

The Third Phase

This vital phase typically lasts 8-16 weeks after surgery; with some protocols, this can last up to 24 weeks. This is the most visible difference in time [20,34]. It constitutes an important landmark in a patient's rehabilitation journey towards normal function and gait [39].

Proprioception and core stability training are frequently suggested in this phase in order to enhance postural control and avoid further instability, particularly in individuals who present with neuromuscular disorders, such as Charcot-Marie-Tooth disease [40,41]. In general, the main aim of phase 3 is to restore gait stability and balance following those complex surgeries. Regular monitoring of circulation, oedema, and neurological status is also emphasized, given the risk of underlying neuropathy or vascular compromise in this patient group [41]. Soft tissue management techniques, such as stretching and protective bracing, are employed to maintain flexibility and reduce the risk of soft tissue contractures during recovery [42,43].

Nonetheless, there are significant disagreements on several phase 3 rehabilitation-related topics. Such disagreements often arise from differing surgical techniques, individual patient healing responses, and institutional philosophies regarding when patients can safely advance to higher levels of activity [34]. Approaches to exercise progression also differ significantly; some programs start with band resistance, while others start with isometric exercises to minimize joint stress in the early stages [27,44]. There is also considerable variability regarding the frequency of physiotherapy, with most institutions recommending regularly scheduled sessions, ranging from one to two sessions a week; others will build their regimen upon the patient's progress and evolution. Personalized physiotherapy protocols should be emphasized, and progression should be based not only on physical milestones but also on patient tolerance and lifestyle [45].

Achieving independent mobility, switching to regular footwear, and maximizing regular movement patterns are the main goals of this phase. Leeuwesteijn et al. emphasized the importance of regaining functional walking ability and restoring a plantigrade foot position to support daily activities [46]. Neumann and Nickisch highlighted that strengthening exercises targeting both the ankle and proximal muscle groups are essential to compensate for neuromuscular imbalances and improve gait mechanics in patients with underlying neurological conditions. The protocols aim to enhance core stability and kinetic chain function while also building grade 4-5 ankle muscular strength [47]. Perera and Guha noted that incorporating proprioceptive training and low-impact cardiovascular activities can help restore dynamic stability while minimizing joint stress during late-stage rehabilitation. Numerous interventions are used to achieve these goals, such as progressive strengthening exercises (from isometric to isotonic), training for balance and proprioception (using BAPS boards and moving on to single-leg activities), and cardiovascular conditioning through walking, swimming, and stationary cycling [48].

Several components of phase 3 rehabilitation require additional research to develop evidence-based best practices. Key areas for inquiry include finding the best time to start specific exercise types, developing evidence-based criteria for phase advancement, and assessing the effectiveness of various manual therapy modalities [49]. Questions remain about the effect of therapy frequency on results, the involvement of biofeedback in muscle activation, and the cost-effectiveness of different treatment frequencies [50]. Furthermore, studies are needed to determine the best progression regimens for strengthening exercises, the benefits of early versus delayed return to normal footwear, the role of hydrotherapy, and the long-term effect of different proprioception training techniques [51].

These significant variations among the protocols, especially with regard to the timelines and progression of exercises, highlight the need for more uniform, evidence-based recommendations and guidelines in this phase [52]. Although there are similarities between the current protocols, variation in specific approaches suggests that best rehabilitation practices may continue to evolve based on clinical experience rather than proven science [53].

The Fourth Phase

Phase 4 is the last phase and usually starts 16 weeks after surgery and may extend up to 12 months, depending on the complexity of the procedure and individual recovery rates [24,54]. This prolonged time reflects the need for bone consolidation, neuromuscular adaptation, and the prevention of recurrence or compensatory gait [54]. Sheean et al. emphasize that custom orthotics, combined with individualized rehabilitation programs, are essential during this phase to restore functional gait and prevent excessive joint stress. Additionally, this phase progresses from basic rehabilitation to sport-specific activities, such as running or advanced functional training, with the aim of restoring higher-level function and enabling patients to return to daily and recreational activities. Regular assessment of limb volume and symptom tracking during this phase can also detect subtle issues, such as adjacent joint overload or late-stage instability, prompting timely adjustments to the rehabilitation plan [38]. All protocols universally agree upon several important points, such as a gradual return to normal footwear, proprioception, balance training, functional strengthening exercises, gait re-education, and maintenance program development. These elements aim to refine gait mechanics, enhance endurance, and build long-term joint stability, especially in patients with previous structural deformities or complex fusions [55,56].

However, there are some circumstances when things change to a great extent. Most importantly, the rehabilitation periods vary significantly among clinicians and facilities. Vier and Ellington observed that this variability is frequently due to variations in the surgical techniques, fixation methods, and patient-related factors, such as bone quality or comorbidities, resulting in varying healing rates and functional milestones [57]. Vertullo and Nunley pointed out that decisions regarding sports were often personalized and took into account the complexity of the surgery, patient expectations, and risk of graft or fusion site stress. In addition to variations in plyometric activity implementation, debate persists on the ideal return-to-sport timing. Some allow running as early as the 20th postoperative week, while others advise not to run for 6-9 months [58]. Another debate is exercise progression, such as methods of introducing challenging exercise and other methods for testing strength. Although with some discrepancy in protocols, some place greater emphasis on early functional strength for exercise progression, while others are more conservative in beginning high-load activities until pain and gait stability focuses are addressed [24,38].

The main goals of this period are as follows: achieve grade 5 muscle power around the ankle, return to usual activities, independent walking without any aids, and implement a lifelong maintenance program. This phase is the peak of the rehabilitation journey and is designed to restore generalized mobility as well as full function and sports readiness where needed [38,58]. Schuh et al. highlighted advanced proprioception and core stability in refining balance and preventing compensatory movement patterns, particularly in patients recovering from complex hindfoot surgeries [56]. Typical interventions include manual therapy techniques, agility training, running progression programs, plyometric training, sport-specific exercises, proprioception training, core stability work, and return-to-sport testing [24,56]. These tests often include strength benchmarks, dynamic balance tests, and functional movement evaluations tailored to the patient's goals and lifestyle demands. Additionally, moving from basic rehabilitation to sport-specific rehabilitation, such as running or advanced functional training, ensures that patients meet certain functional criteria before engaging in high-demand activities, thereby minimizing the risk of re-injury or fusion site stress [24,38].

**Figure 2:**
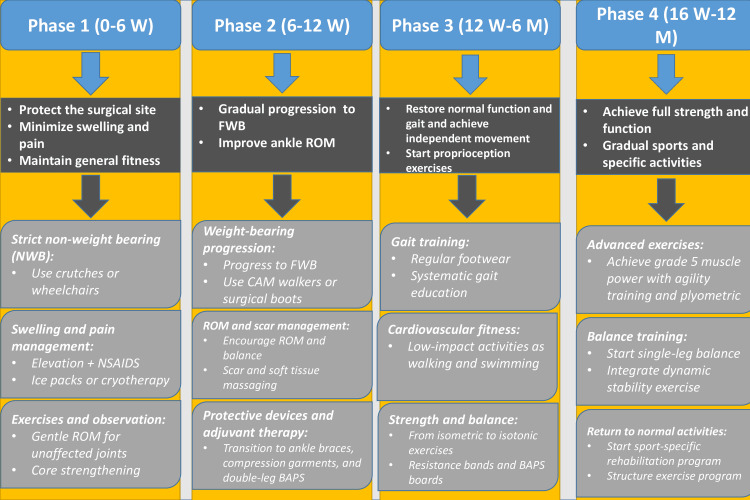
Postoperative rehabilitation protocol for calcaneal osteotomy, subtalar fusion, and triple fusion NSAIDS: non-steroidal anti-inflammatory drugs; ROM: range of motion; FWB: full weight-bearing; CAM: controlled ankle motion; BAPS: Biomechanical Ankle Platform System Image Credits: Ahmed Shalaan

There are several research gaps in this area with higher research needs. These include the most appropriate timing for starting running programs, evidence-based criteria for return to sport, the effectiveness of plyometric progression protocols, and the effects of different timings on long-term outcomes. Addressing these knowledge gaps is crucial not only for improving the outcomes of this surgery but also, first, for optimizing patients' satisfaction with this procedure and, second, for safely accelerating one's return to the previously desired activities [57,58] (Figure 2).

Factors influencing protocol selection and variations among different protocols

**Figure 3:**
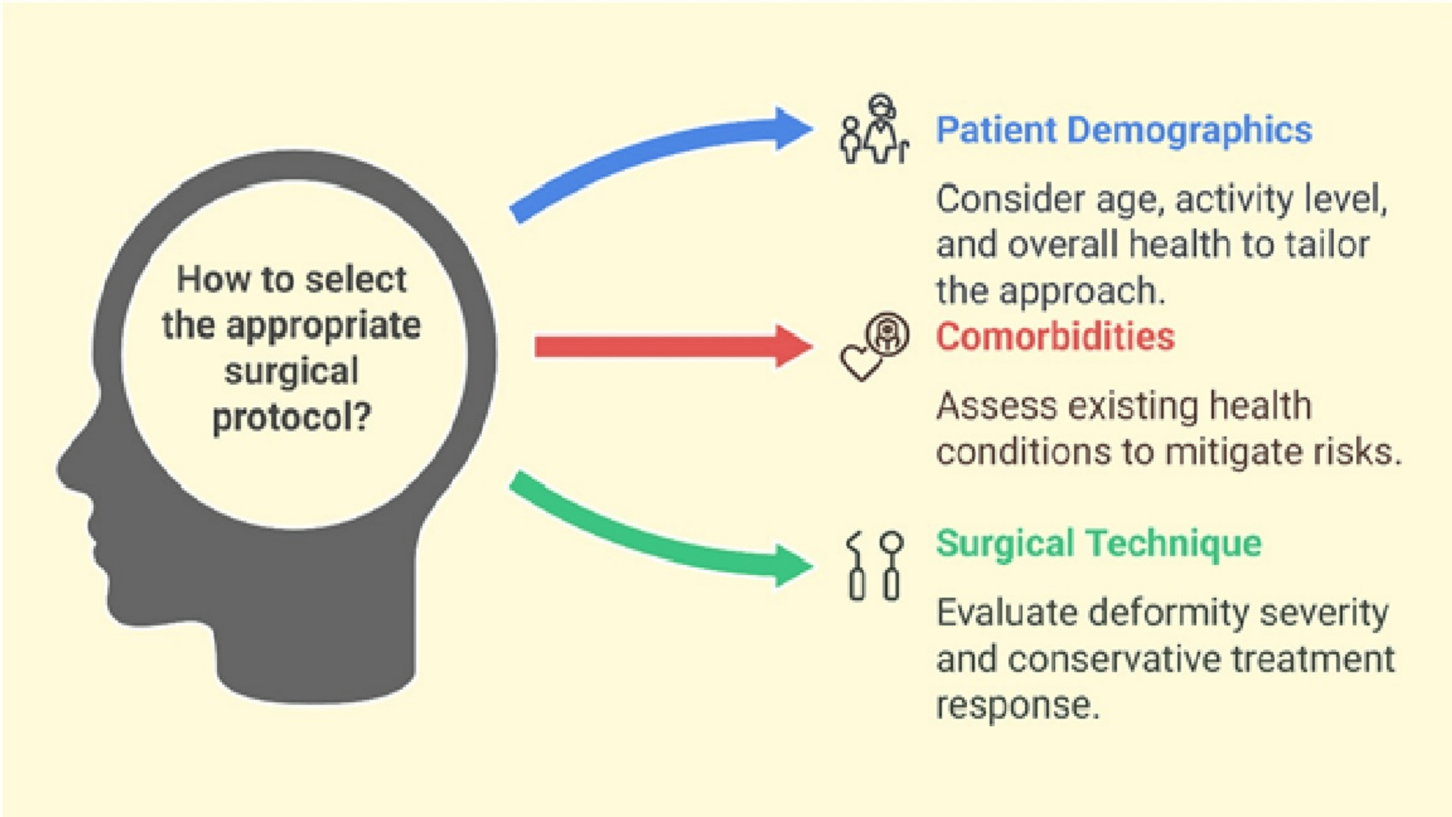
Factors influencing protocol selection and variations among different protocols Image Credits: Ahmed Shalaan

Protocol selection depends on patient demographics and comorbidities and the characteristics of the surgical intervention. Patient factors, including age, functional demand, and comorbidity, significantly influence the management plan and the subsequent rehabilitation. Surgical procedures in younger, more active patients tend to focus on joint preservation and long-term function restoration, whereas in older patients, or those who are less active, procedures may be less technically complex and more focused on pain relief and joint stability [59]. Comorbidities like diabetes, cardiovascular disorders, and peripheral vascular disease should also be kept in mind, as they may influence infection rates and the healing process. Wiewiorski et al. noted that patients with multiple comorbidities are at a much higher risk for wound complications, which may strongly affect the choice of the surgical approach and the postoperative rehabilitation program [60]. Additionally, the choice of surgical technique is influenced by the degree of deformity, arthrosis, and the patient's response to conservative treatments. Thordarson highlighted that procedures like triple fusion are often reserved for severe, rigid deformities or advanced arthritic changes, whereas less invasive options such as calcaneal osteotomy or isolated subtalar fusion may suffice for less complicated cases [61]. Each patient's characteristics guide the surgeon in choosing the most effective management, either conservatively or surgically, and hence this will certainly reflect on their rehabilitation protocol. In summary, individualized care according to the balance between surgical goals and the levels of patient-specific risks is needed to maximize postoperative recovery and functional success. Although such individualized protocols are necessary in real-world care, much about postoperative rehabilitation remains poorly evidence-based. Consequently, there is a demand for well-conducted research to help fill these lacunae and provide more robust information for clinical decision-making (Figure 3).

Key priorities for future research in hindfoot surgery

**Figure 4:**
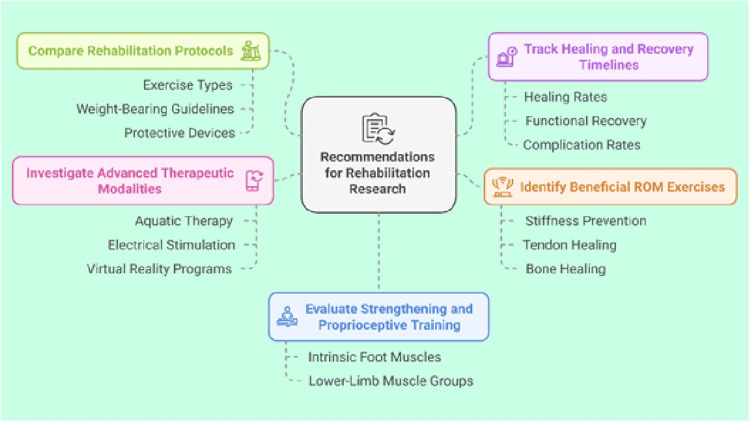
Key priorities for future research directions ROM: range of motion Image Credits: Ahmed Shalaan

In light of the factors influencing protocol selection, it is evident that current research remains inconclusive, highlighting the need for more robust evidence to establish a standardized postoperative rehabilitation protocol. We recommend conducting additional large-scale, multicenter randomized controlled trials with high methodological quality, extended follow-up periods, and well-defined outcome measures (Figure 4). These studies should be prioritized to first address the most fundamental and variable aspects of care:

Highest Priority: Core Rehabilitation Components

(a) Compare distinct rehabilitation protocols to identify which specific components, such as exercise types, weight-bearing guidelines, or use of protective devices, optimize functional outcomes, pain control, and overall recovery. (b) Track healing rates, functional recovery, and complication rates to determine the safest and most efficient timelines for initiating partial and full weight-bearing and clarify how early or delayed starts influence long-term results. (c) Identify the most beneficial ROM exercises and the ideal time to begin them, focusing on preventing stiffness, enhancing tendon and bone healing, and promoting a stable return to daily activities.

Medium Priority: Personalization and Standardization

(a) Evaluate strengthening exercises and proprioceptive training methods that best restore function and balance, focusing on intrinsic foot muscles and larger lower-limb muscle groups. (b) Adopt consensus-driven strategies to establish standardized outcome metrics for evaluating recovery. This includes identifying barriers and facilitators that affect the implementation of uniform protocols in diverse clinical settings. (c) Develop individualized rehabilitation plans that incorporate patient-specific factors such as age, comorbidities, and surgical details. Predictive modelling could further refine these plans by identifying key indicators for optimal outcomes. (d) Address the psychological aspects of recovery, including anxiety, depression, and motivation, and their impact on rehabilitation adherence and outcomes. Incorporating patient-reported outcome measures (PROMs) will be critical to capturing the full patient experience and tailoring interventions accordingly.

Innovative Priority: Advanced and Supportive Modalities

(a) Investigate advanced therapeutic modalities, including aquatic therapy, electrical stimulation, and virtual reality-based programs, to assess their effectiveness in accelerating recovery and improving patient engagement. (b) Perform cost-effectiveness analyses to determine which protocols offer the most favorable balance of outcomes and resource utilization, aiding decision-makers in both clinical and administrative contexts. (c) Examine new technologies, such as mobile applications, wearable sensors, and telerehabilitation platforms, to ascertain their efficacy in monitoring patient progress and improving adherence to prescribed protocols.

## Conclusions

Effective management of foot deformities through calcaneal osteotomy, subtalar fusion, and triple fusion relies on precise surgical techniques and robust postoperative rehabilitation protocols. The recommended protocols emphasize gradual weight-bearing, tailored ROM exercises, and patient-specific strategies to enhance healing and functional recovery. Patient compliance is pivotal in achieving optimal outcomes, minimizing complications, and ensuring stable joint function. However, the variation across current protocols highlights the urgent need for standardized guidelines.

This review consolidates key rehabilitation principles across different hindfoot procedures, giving clinicians a clearer understanding of current practices and their limitations. Clinicians are encouraged to apply these insights in daily practice, while researchers are urged to develop large-scale, high-quality studies that can generate consensus-based protocols. Such work will be essential to translate current variability into evidence-based, universally accepted standards that improve both functional recovery and long-term patient satisfaction.
